# The community-curated *Pristionchus pacificus* genome facilitates automated gene annotation improvement in related nematodes

**DOI:** 10.1186/s12864-021-07529-x

**Published:** 2021-03-25

**Authors:** Christian Rödelsperger

**Affiliations:** Department for Integrative Evolutionary Biology, Max Planck Institute for Developmental Biology, Max-Planck-Ring 9, 72076 Tübingen, Germany

**Keywords:** Comparative genomics, Evolution, Phylogeny, Parasite, *Caenorhabditis elegans*, BUSCO, PPCAC

## Abstract

**Background:**

The nematode *Pristionchus pacificus* is an established model organism for comparative studies with *Caenorhabditis elegans*. Over the past years, it developed into an independent animal model organism for elucidating the genetic basis of phenotypic plasticity. Community-based curations were employed recently to improve the quality of gene annotations of *P. pacificus* and to more easily facilitate reverse genetic studies using candidate genes from *C. elegans*.

**Results:**

Here, I demonstrate that the reannotation of phylogenomic data from nine related nematode species using the community-curated *P. pacificus* gene set as homology data substantially improves the quality of gene annotations. Benchmarking of universal single copy orthologs (BUSCO) estimates a median completeness of 84% which corresponds to a 9% increase over previous annotations. Nevertheless, the ability to infer gene models based on homology already drops beyond the genus level reflecting the rapid evolution of nematode lineages. This also indicates that the highly curated *C. elegans* genome is not optimally suited for annotating non-*Caenorhabditis* genomes based on homology. Furthermore, comparative genomic analysis of apparently missing BUSCO genes indicates a failure of ortholog detection by the BUSCO pipeline due to the insufficient sample size and phylogenetic breadth of the underlying OrthoDB data set. As a consequence, the quality of multiple divergent nematode genomes might be underestimated.

**Conclusions:**

This study highlights the need for optimizing gene annotation protocols and it demonstrates the benefit of a high quality genome for phylogenomic data of related species.

**Supplementary Information:**

The online version contains supplementary material available at 10.1186/s12864-021-07529-x.

## Background

Genome sequencing efforts across all domains of life have broadened our understanding about how phenotypic novelty coincides with genomic innovations. This was facilitated by continuous improvement of sequencing technology during the last two decades, which allowed the generation of high-quality genome assemblies in large-scale phylogenomic contexts. In contrast, gene annotation protocols have evolved at a much slower rate [1]. One reason for the slow progress in the further developments of gene annotation protocols was likely the absence of universal benchmarking standards. Even though gene predictions were often evaluated against available expression data, such results were difficult to compare between organisms due to strong differences in transcriptomic resources across various genome projects. This changed when alternative approaches, such as benchmarking universal single copy orthologs (BUSCO) [2], employed comparative genomics to define a set of highly conserved orthologous genes that should be expected in a given genome assembly. While the BUSCO completeness level has become a widely used quality measure with similar importance as the N50 measure for assembly contiguity, its informative value is highly dependent on the quality and sampling of the underlying orthology data, which may differ vastly across taxonomic groups. Currently, multiple representative genomes of the highly diverse and rapidly evolving nematode phylum are still poorly annotated. To overcome this problem in the case of the nematode model organism *Pristionchus pacificus*, community-based curations have recently been initiated to improve the quality of gene annotations [3, 4]. *P. pacificus* was initially established for comparative studies with the classical nematode model organism *Caenorhabditis elegans* [5], but more recently it gained importance as an independent model system for elucidating the genetic basis of phenotypic plasticity [6–8] and the emergence of novel genes [9–11]. *P. pacificus* has a chromosome-scale genome assembly and computationally generated gene annotations based on transcriptomic data, protein homology data, and gene predictions were of relatively high quality [12]. However, further strand-specific RNA-seq and Iso-seq data pointed towards the presence of numerous artificial gene fusions in gene dense regions of the genome [3]. This motivated a screen for suspicious gene models based on comparative genomic approaches and to propose corrections after manual inspection by community annotators. Two rounds of community curations improved the BUSCO completeness level from 86 to 98% (nematode odb9 data set) [2–4].

Here, I make use of the community-curated annotations of the *P. pacificus* genome to improve the annotations of related *Pristionchus* and other genomes of the family Diplogastridae, which were recently sequenced to study the evolutionary dynamics of novel gene families [9]. This demonstrates that a single high quality reference data set is sufficient to improve gene annotations in related genomes.

## Results

### High quality gene annotations are rare outside the *Caenorhabditis* clade

**Fig. 1:**
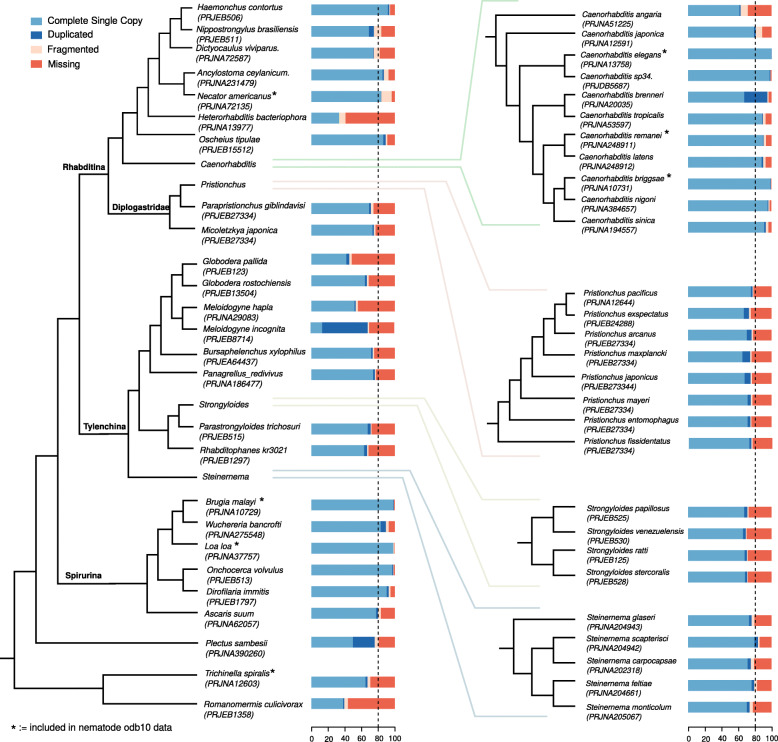
Comparative analysis of gene annotation quality based on BUSCO completeness. Gene annotations from 54 nematodes were retrieved from WormBase ParaSite (WBPS14) and quality was assessed based on the BUSCO approach (nematode odb10). The bar charts indicate the percentage of proteins falling into a specific BUSCO class. The schematic species trees are redrawn from Smythe et al. and other phylogenomic studies [9, 14–17]

In order to assess the current status of nematode genome quality, I analyzed gene annotations from 54 nematode species as obtained from WormBase ParaSite (version WBPS14) using the BUSCO approach, which tests for the presence of highly conserved single copy orthologs [2, 13]. Using an arbitrary cutoff of > 80% BUSCO completeness of single copy genes to define high quality gene annotations, this analysis shows that high quality gene annotations are rare outside the *Caenorhabditis* clade (Fig. 1). Few exceptions are the genomes of the free-living *Oscheius tipulae* and the parasitic *Haemonchus contortus* [18], *Dirofilaria immitis* [19], *Loa loa* [20], *Brugia malayi* [21], and *Onchocerca volvulus* [22]. Please note that the most recent updates of the *P. pacificus* gene annotations have not yet been integrated into WormBase, which explains why the BUSCO completeness is shown at around 80% (Fig. 1). As BUSCO genes are defined as genes that should be present as single copy in at least 90% of genomes, the low completeness values point towards substantial annotation problems in various genomes (Fig. 1). Alternatively, these discrepancies could reflect true cases of gene losses in specific lineages. Another explanation could be that divergent orthologs may not be detected, as the underlying nematode odb10 data set only contains seven nematode species (Fig. 1) and these do not represent the full range of genomic diversity of the nematode phylum [14, 23]. Despite these alternative explanations, the phylum-wide assessment of annotation quality based on BUSCO completeness strongly suggests substantial need for improvement, which is in accordance with complementary studies assessing the quality of multiple nematode genomes [24, 25].

### A single high quality reference set is sufficient to improve gene annotations in related species

**Fig. 2:**
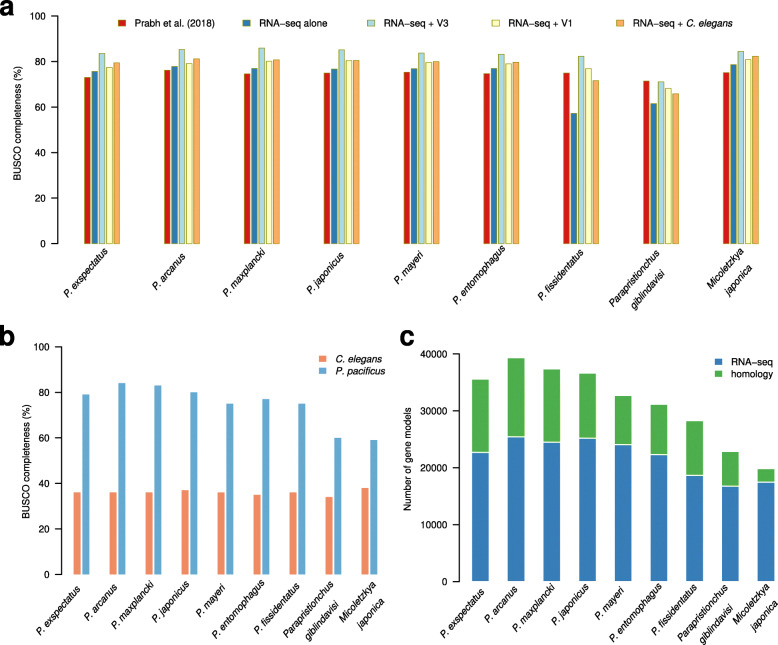
Improved gene annotations of nine diplogastrid genomes. **a** Reannotation of nine diplogastrid genomes based on available RNA-seq data and the community-curated *P. pacificus* gene annotations results in a median improvement of 9% BUSCO completeness (single copy + duplicated, nematode odb10) over the previous versions [9]. **b** The barplots show the BUSCO completeness of annotated diplogastrid genomes, when only homology data from either *C. elegans* or *P. pacificus* are used. The more closely-related *P. pacificus* gene annotations are a consistently better reference data set than the *C. elegans* data. However, the quality of annotations inferred from homology data seems to decrease considerably when divergence exceeds the genus level. **c** The stacked barplot shows the number of gene models derived from RNA-seq or homology data for the final annotations

Previously, comparative genomic screens were combined with community curation to improve the quality of gene annotations in *P. pacificus* [4]. Here, the objective is to demonstrate how community curation of a single genome can be used to automatically improve the quality of phylogenomic data from related species. To this end, I reannotated nine nematode genomes of the family Diplogastridae including seven other *Pristionchus* species, which were sequenced previously as part of a phylogenomic study to investigate the evolutionary dynamics of novel gene families [9, 26]. Specifically, predicted open reading frames (ORFs) in assembled RNA-seq transcripts [27] as well as protein sequences of the community-curated *P. pacificus* annotation (El Paco gene annotation, version 3) were mapped to the draft genomes with the help of the exonerate alignment tool [28]. Subsequently, a simple heuristic was applied to pick only one representative gene model per locus (see *Methods*, Additional file 1, Figure S1). The raw mappings of transcribed ORFs, the previous and the newly generated gene annotations were then evaluated based on the level of BUSCO completeness (nematode odb10 data set). To assess, to what extent the manually improved *P. pacificus* data set yielded better gene annotations of related nematodes, the same reannotation procedure was applied using the automatically generated *P. pacificus* gene annotations as homology data [12]. Similarly, the gain in gene annotation accuracy by having a more closely related reference data set was evaluated by comparison with the highly curated but evolutionary distant *C. elegans* annotation. For the nine diplogastrid genomes, reannotation with the community-curated *P. pacificus* genome yielded a median BUSCO completeness of 84% (Fig. 2a, Table 1), which corresponds to a median improvement of 9% over the previous version of gene annotations [9]. The genome of *Parapristionchus giblindavisi* is the only exception where the new *P. pacificus* annotations did not result in an improved gene annotation (Fig. 2a). Most likely, this is due to general problems with this assembly, as it has the lowest level of contiguity, a high fraction of ambiguous bases, and the highest ratio of incorrectly oriented read pairs in realignment analysis [9]. Such problems potentially arose from remaining heterozygosity [29] and might impair accurate gene annotation. However, all other genomes showed that using the community-curated *P. pacificus* data set as homology data yielded the most complete gene annotations even when compared to the highly curated but evolutionary distant *C. elegans* data set (Fig. 2a).

**Table 1:** Assembly and gene annotation features of diplogastrid genomes

Species	Prabh et al. 2018			This study		
	Number of gene models	BUSCO odb9 C/D/F/M (%)	BUSCO odb10 C/D/F/M (%)	Number of gene models	BUSCO odb9 C/D/F/M (%)	BUSCO odb10 C/D/F/M (%)
*P. exspectatus*	31,172	79/7/6/8	67/6/2/25	35,595	85/7/5/4	77/7/2/14
*P. arcanus*	35,909	81/7/4/8	71/6/2/22	39,331	86/8/3/2	79/7/2/13
*P. maxplancki*	31,765	75/11/7/8	65/7/2/23	37,393	80/13/4/2	74/12/2/12
*P. japonicus*	31,996	77/8/6/10	68/7/2/23	36,638	84/9/4/4	76/9/2/13
*P. mayeri*	36,554	81/5/6/8	71/4/2/23	32,719	86/5/6/4	79/4/3/14
*P. entomophagus*	37,279	82/4/6/8	71/3/1/24	31,150	87/4/5/4	79/4/2/15
*P. fissidentatus*	25,634	83/2/6/10	73/2/2/24	28,283	87/2/7/4	80/3/3/15
*P. giblindavisi*	35,770	74/3/10/13	69/2/3/26	22,872	70/3/14/13	68/3/6/23
*M. japonica*	24,971	79/2/9/9	73/2/2/23	19,855	88/2/5/5	82/2/2/13

The table shows a comparison between the previous [9] and the current gene annotations for nine diplogastrid genomes. The number of gene models is denoted together with the BUSCO results for the odb9 (*N* = 982 orthologs) and odb10 (*N* = 3131 orthologs). *C* Complete single copy, *D* Duplicated, *F* Fragmented, *M* Missing

### Divergence across genera hinders gene model inference from homology data

The fact that homology data from *P. pacificus* yields better results than *C. elegans* data indicates that the ability to transfer gene annotations across species drops with increasing sequence divergence. This would imply that taking the highly curated *C. elegans* data as a reference, is suboptimal for annotating divergent nematode genomes. To test if the drop in homology-based annotation accuracy occurs already within the same nematode family, I reevaluated the BUSCO completeness of the gene annotations only inferred from homology data (Fig. 2b). While gene models based on homology with *C. elegans* have a constantly low BUSCO completeness of around 36%, the completeness values for the gene models inferred from community-curated *P. pacificus* data range between 59 and 84% (Fig. 2b). Note that the two non-*Pristionchus* genomes have with 59 and 60% the lowest completeness values whereas the lowest value for the remaining *Pristionchus* genomes is 75% (Fig. 2b). Moreover, the evaluation of the contribution of homology-inferred vs. RNA-seq derived gene models to the final gene annotations also show a drop in the contribution of homology data for the two non-*Pristionchus* species (Fig. 2c). Taken together, these results suggest that the ability to transfer gene models based on protein conservation already drops beyond genus-level sequence divergence. This strongly limits the usefulness of model organism data such as from *C. elegans* to be helpful for annotating genomes in evolutionary distant nematode clades.

### The quality of several nematode genomes might be underestimated

**Fig. 3:**
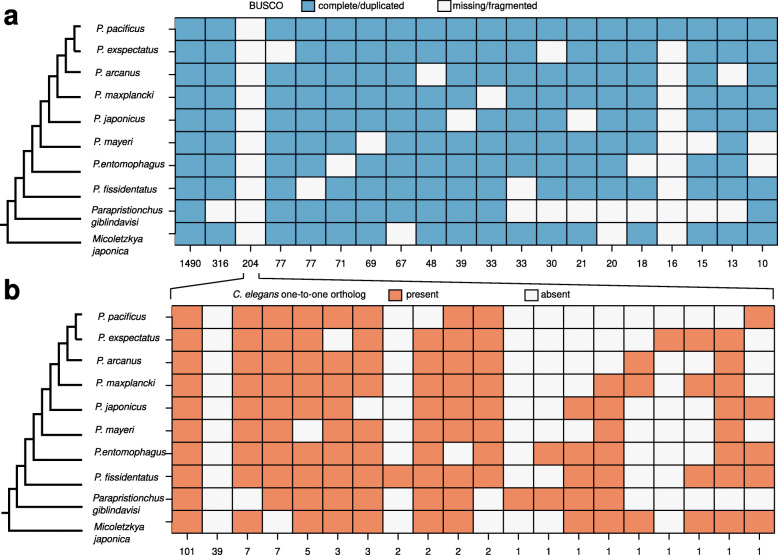
Missing BUSCO orthologs in diplogastrid genomes. **a** Classifications from the BUSCO results (nematode odb10) were summarized across all diplogastrid genomes and the twenty most abundant patterns are displayed. While misannotations should result in random patterns with low frequency, highly abundant patterns can point towards problematic data sets (e.g. *Parapristionchus*), true biological events (e.g. gene losses) or systematic biases. **b** Complementary ortholog predictions based on best-reciprocal BLASTP hits show that 101 (49.5%) of BUSCO genes, which were predicted to be missing in all diplogastrid genomes, have putative one-to-one orthologs

**Fig. 4:**
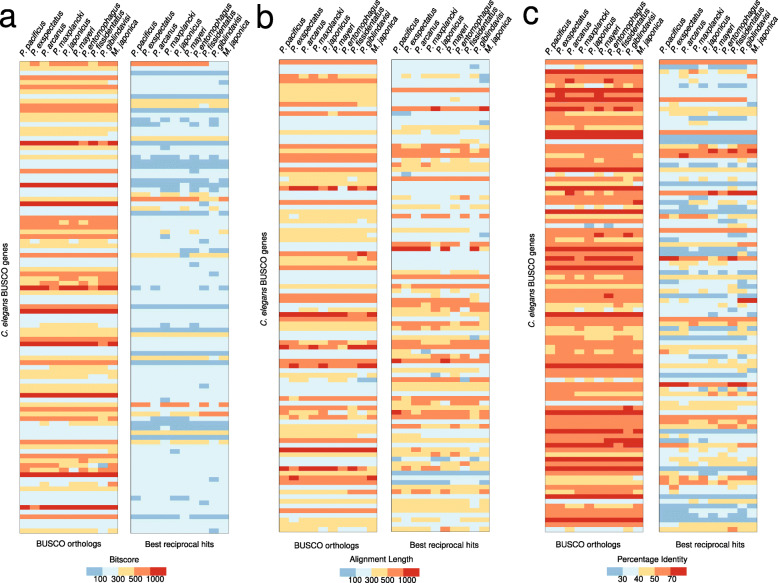
Sequence divergence causes orthology detection failures. **a** The left heatmap shows the bitscores for 101 randomly subsampled BUSCO orthologs derived from a BLASTP search of the *C. elegans* proteins against annotated protein sets of the ten diplogastrid genomes. The right heatmap shows the bitscores for 101 putative orthologs that were only predicted based on best-reciprocal BLASTP hits. **b** The heatmaps show the BLASTP alignment lengths for BUSCO genes from *C. elegans* against diplogastrid proteins for 101 randomly subsampled genes with BUSCO orthologs (left) and for 101 best-reciprocal hits (right). **c** The heatmaps show the percentage identity between BUSCO genes from *C. elegans* and their putative diplogastrid orthologs

**Fig. 5:**
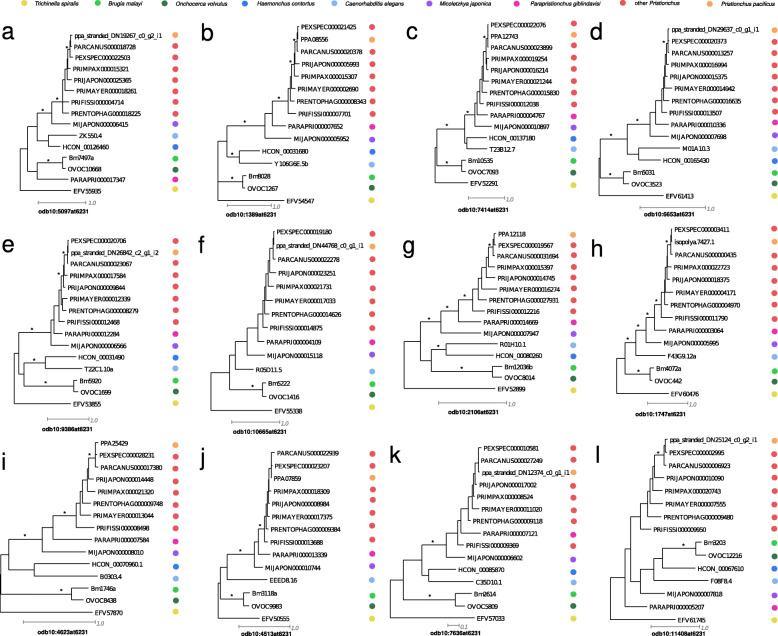
Phylogenetic analysis supports orthology relationships. Twelve candidate gene families (**a**-**l**), where BUSCO failed to detect orthologs in diplogastrid genomes, were selected as candidates for phylogenetic analysis. Maximum-likelihood trees were reconstructed from BUSCO orthologs of other nematode species as well as diplogastrid sequences, which were identified as best-reciprocal hits against *C. elegans* proteins. Stars denote branches with a bootstrap support ≥99/100. In most cases, the phylogenies are largely consistent with the species tree and the diplogastrid sequences form a monophyletic clade that correctly groups as sister to the rhabditids (*C. elegans* and *H. contortus*)

The new gene annotations of the nine diplogastrid genomes contain between 19 and 39 thousand gene models that are completely evidence-based as they are either supported by transcriptional evidence or by protein conservation with *P. pacificus* (Table 1). However, between 9 and 15% of BUSCO genes (nematode odb10 data set) seem to be missing in the *Pristionchus* genomes (Table 1). If these missing genes are due to misannotations in individual genomes, further rounds of manual corrections across this phylogenomic data set could be used to reannotate missing genes in selected genomes. In such a case, the abundance of gene absence/presence patterns should be more or less randomly distributed. However, if missing genes are due to massive gene losses across the diplogastrid lineage, the distribution of patterns should be dominated by a phylogenetic pattern that could be parsimoniously explained by a single evolutionary event (Fig. 3a). The two most abundant presence/absence patterns are 1490 BUSCO genes that are found in all nine diplogastrid genomes and 316 genes that seem to be missing in the *Parapristionchus giblindavisi* genome, which seems to be the most problematic data set (Fig. 3a). The third most abundant pattern arises from 204 genes that were not found in any of the diplogastrid genomes, which is suggestive of a lineage-specific gene loss. To exclude that these genes are missing due to gene annotation failure, the BUSCO pipeline was run in genome mode against the raw assembly (Additional file 1, Table S1). This confirmed that 185 (91%) of those genes could not be detected by running BUSCO on the raw genome assembly. As mentioned above, an alternative explanation would be that these genes are present but could not be detected by the BUSCO pipeline as the nematode odb10 data set does not represent the full diversity of the nematode phylum [14, 23]. Thus, running the BUSCO pipeline with a phylogenetically more broadly sampled set of taxa should capture these missing genes. Consistently, the older nematode odb9 data set, which included data from *P. pacificus*, yields higher completeness values (median value 91%, Table 1). To further test the possibility of undetected orthologs, I used a complementary approach to find one-to-one orthologs of the corresponding *C. elegans* genes in the diplogastrid genomes based on best reciprocal BLASTP searches. This revealed that 101 (50%) of these 204 genes have predicted one-to-one orthologs in all diplogastrid genomes, which points to a failure of detection of the BUSCO pipeline (Fig. 3b). Comparison of the bitscores from BLASTP searches between BUSCO genes in *C. elegans* and their putative orthologs in the diplogastrid genomes shows a pronounced difference between the diplogastrid sequences that were detected as orthologs by BUSCO and the sequences that were only identified as best-reciprocal hits (Fig. 4a). Further analysis of alignment length and percentage identity indicates that this difference is due to stronger sequence divergence in the diplogastrid sequences that were not identified as orthologs by BUSCO (Fig. 4b, c). Similarly, the analysis of length-normalized bitscores shows strong differences between BUSCO orthologs and best-reciprocal hits, whereas the overall aligned proportion is more comparable between both groups (Additional file 1, Figure S2). To further support that at least some of these genes are truly orthologs, I reconstructed gene trees for 12 randomly chosen candidate gene families (Fig. 5a-l). Most of these phylogenies largely resemble the species phylogeny (Fig. 1) and therefore support the one-to-one orthology relationship. Thus, I conclude that the insufficient sample size and phylogenetic breadth of the nematode odb10 data set may cause failures in the ortholog detection by the BUSCO pipeline and that the quality of divergent nematode genomes might therefore be underestimated.

## Discussion

In this study, I have demonstrated the benefit of the community-curated *P. pacificus* gene annotations for phylogenomic data of related species. The automated improvements of gene annotations in other diplogastrid nematodes suggest that not every genome has to be manually curated, but that a single high-quality genome per genus or family is sufficient to allow effective gene model inference based on homology. It has to be noted that community curation is not the only way to obtain high quality gene annotations as previous studies showed that high levels of BUSCO completeness could be obtained by extensively optimized application of gene prediction tools such as BRAKER and AUGUSTUS [25, 30, 31]. Specifically, the training of gene models is the most important step for such ab initio gene prediction methods. However, the question how good gene annotations have to be will depend on the research topics that are going to be studied. Previous studies of novel gene origin in *P. pacificus* pointed towards an inflated number of species-specific orphan genes that are most likely gene prediction artifacts recognizing spurious coding signals on the antisense strand of truly protein-coding genes [10]. Thus, to facilitate more conclusive studies of gene birth processes using purely evidence-based set of gene annotations, species-specific genes without RNA-seq support were removed from current annotations [4]. Furthermore, other types of gene prediction artifacts such as artificially fused gene models do not necessarily impact BUSCO completeness levels but impair the detection of orthologs [3]. In this context, community annotation seems to be one of the most effective methods to increase gene annotation quality beyond what can be achieved using automated pipelines. For example, in the case of the nine diplogastrid genomes, BUSCO genes that are found in the genome or transcriptome (Additional file 1, Tables S1, S2), but not in the final gene annotations can be taken as candidates to further improve gene annotation quality by manual curation. Nevertheless, it has to be noted that the effectiveness of community annotation is highly dependent on the availability of abundant transcriptomic resources. Thus, requiring support by either transcriptome data or protein conservation for every gene is not generally feasible for genome projects of phylogenetically isolated species with limited expression data.

The drop in the ability to infer gene models based on homology beyond the genus level emphasizes the rapid evolution and extreme diversity of nematode lineages [32, 33]. This might also at least partially contribute to the failure of detection of 101 putative orthologs by the BUSCO pipeline, as the underlying OrthoDB data set (nematode odb10) appears to be too small and unevenly sampled to capture the full diversity of the nematode phylum [14, 23]. As a consequence, the quality of many nematode genomes might be underestimated. In the case of the diplogastrid genomes, the completeness level is likely underestimated by up to 3% (101 out of 3131 BUSCO genes that were not detected but are present in all 10 genomes). The fact that this problem seems to be less pronounced in the older odb9 data set (Table 1) suggests that the more recent odb10 data set is not well suited for assessing the genome quality of divergent nematode species.

Thus, my study not only highlights the need to optimize gene annotation pipelines, but also points at potential problems in the benchmarking processes. Further work will be needed to establish more comprehensive benchmarking data sets as well as to optimize annotation protocols. For example, apart from the alignment program exonerate [28], there are multiple alternative approaches for transferring gene models such as GMAP or liftover, and Liftoff [34, 35]. In addition, gene models from transcriptome and homology data were integrated into a non-redundant gene set using a heuristic approach that selects the gene model with longest ORF per 100 bp window. Hereby, genes with less than three exons or a predicted protein length of less than 60 amino acids were discarded. Such arbitrary thresholds may remove unusually short genes with important biological functions and might not be directly transferable to other nematode genomes with a smaller number of exons per gene than *P. pacificus* [36]. Apart from the mentioned caveats, the presented methods should be directly applicable to genomes of other taxonomic groups, even outside of nematodes.

In future, gene model inference based on the highly curated *P. pacificus* data set will help other genomic studies in this clade of nematodes. Currently, the genus *Pristionchus* has around 50 described species and genus-wide phylogenetic studies revealed interesting trends such as the parallel emergence of hermaphroditism and the convergent evolution of specific pheromones [27, 37]. The generation of high quality genomes of further members of the *Pristionchus* genus may therefore help to characterize and compare the genomic basis of these convergent patterns. Simultaneously, the pool of more than thousand *P. pacificus* strains was successfully exploited to dissect the genetic basis of phenotypic variation at a population level [38, 39]. Such unbiased genetic screens revealed that genomic changes including gene duplication and loss cause natural variation in pheromone production and response [40, 41]. Thus, properly annotated de novo assemblies of different *P. pacificus* strains will greatly aid the interpretation of associations between genotypes and phenotypes and thus complement future genetic screens in *P. pacificus*.

## Conclusions

While genome sequencing technologies have undergone tremendous development over the past 20 years, gene annotation protocols evolved at a much slower rate. In the case of the nematode model organism *Pristionchus pacificus*, community-based gene curations have previously been presented as an effective means to lift annotation quality above the level of what could be obtained by automated pipelines. Here, I make use of these community-curated annotations to automatically improve phylogenomic data of nine related nematodes. This work has three major conclusions. First, the community-curated *P. pacificus* genome improves the completeness of related nematode genomes by a median of 9% over previous annotations. With BUSCO completeness levels between 83 and 86%, the reannotated *Pristionchus* genomes are more complete than most other members of the nematode phylum. Second, the ability to infer gene models based on homology already drops beyond the genus level, which implies that the highly curated *C. elegans* data is not well suited for annotation of divergent nematode genomes. Third, the insufficient sample size and phylogenetic breadth of the BUSCO and OrthoDB data sets may prohibit the detection of orthologs and thus cause an underestimation of nematode genome quality.

## Methods

### Generation of evidence-based gene annotations

The data set of transcribed ORFs was taken from a previous study where transcriptomes of different Diplogastrid species were assembled from mixed-stage RNA-seq data (Additional file 1, Table S2) and partial and complete ORFs with a minimal length of 40 amino acids were extracted [27]. These transcribed ORFs were aligned against the respective reference assembly with the help of the exonerate protein2genome program with the following parameter settings: --bestn 2, −-dnawordlen 20, and --maxintron 20,000 (version 2.2.0) [28]. The homology model data set was generated by aligning *P. pacificus* proteins (El Paco gene annotation, version 3) against the reference assemblies using exonerate with the same parameter settings [4]. The -bestn option was set to two in order to annotate potential duplicates. Homology models and transcribed ORFs were merged into a joint annotation which included possibly multiple gene models (different isoforms in the assembled transcripts, different evidence types) for a given gene. The complexity of the joint annotation was reduced by a simple heuristic to generate a set of non-redundant annotations. First, gene models from each DNA strand were separated and every start and end coordinate of an exon was assigned to a 100 bp window. Second, For each 100 bp window the coverage was computed as the number of features that were assigned to this window. Third, starting with the most highly covered 100 bp window, the intersecting gene model with the longest ORF was chosen whereas all other intersecting gene models were discarded. At this step only gene models with ORFs of at least 60 amino acids and at least three exons were considered. This last step was successively executed for all other windows. As gene models in *P. pacificus* tend to have more exons per gene than most other nematodes [36], the threshold of at least three exons per genes was implemented to prevent an inflation of gene counts by partially assembled transcript fragments and transcriptional noise. The source code for generating these gene annotations is written in perl. All scripts have been compiled in a software package called PPCAC and are available at https://github.com/roedelsberg/ppcac/.

### Comparative genomic analysis

Protein sequences from 54 nematodes were retrieved from WormBase ParaSite (WBPS14). In case of multiple isoforms, the longest isoform was chosen as the representative sequence [20, 33, 42–58]. To assess the completeness level of gene annotations, the BUSCO pipeline (version 4.1.1) was run in protein mode with the nematode odb10 data set (Creation date: 2019-11-20, number of species: 7, number of BUSCOs: 3131). This combination of BUSCO and odb10 was used for most analyses (Figs. 1, 2 and 3). However, for comparisons with a set of orthologous genes, which include data from *P. pacificus*, the BUSCO pipeline (version 3.0.1) was run with the nematode odb9 data set (Creation date: 2016-02-13, number of species: 8, number of BUSCOs: 982). From the result files of the BUSCO pipeline, genes that were classified to be missing were extracted and compared with predicted one-to-one orthologs for *C. elegans* genes that were obtained by best-reciprocal BLASTP searches (version 2.6.0, e-value < 0.0001, bitscores were used to define the best BLASTP hit). From the BLASTP results, bitscores, percentage identity, and alignment lengths were extracted and used for comparison between the BUSCO orthologs and best-reciprocal hits. For visualization and comparison of BLASTP features, the set of 1490 BUSCO genes with orthologs in all diplogastrid genomes (Fig. 3a) was repeatedly downsampled to match the 101 putative orthologs that were identified by best-reciprocal BLASTP searches (Fig. 3b). Phylogenies of selected orthologous groups were generated by extracting the corresponding BUSCO orthologs in the high quality gene annotations of *C. elegans*, *Haemonchus contortus*, *Brugia malayi*, *Onchocerca volvulus, and Trichinella spiralis* and combining these with the best-reciprocal hits in the diplogastrid genomes. Subsequently, protein sequences were aligned by the MUSCLE program (version 3.8.31, default options) and maximum likelihood trees were calculated using the phangorn package in R (version 3.4.4, LG substitution model with optimization of base frequencies and invariant sites, 100 bootstrap pseudoreplicates) [59, 60]. The orthologous *T. spiralis* sequences, as identified by BUSCO pipeline, were used to root the trees.

## Supplementary Information

**Additional file 1: Figure S1.** Heuristic approach for reducing the complexity of redundant annotations. A 30kb genomic locus is visualized in the Integrative Genomics Viewer with different tracks showing the exonerate alignments of protein homology data, transcribed ORFs, and the resulting non-redundant gene annotations. The lower plot shows the coverage of exonic features in 100bp windows. First, a 100bp window with maximal coverage is selected. Second, the overlapping gene model with longest ORF is chosen as representative gene model for this locus. Third, all other overlapping gene models are excluded from further analysis. Fourth, the next 100bp window is chosen. This procedure is continued until all 100bp windows have been processed. The final gene models are shown in the track labeled as ‘Non-redundant gene models’. **Figure S2.** Comparison of normalized bitscores and aligned proportion. a The left heatmap shows the normalized bitscores (bitscore / alignment length) for 1490 BUSCO orthologs derived from a BLASTP search of the *C. elegans* proteins against annotated protein sets of the ten diplogastrid genomes. The central heatmap shows the data for 101 randomly subsampled BUSCO orthologs. The right heatmap shows the normalized bitscore for 101 putative orthologs that were only predicted based on best-reciprocal BLASTP hits. b The aligned proportion was computed as the length of the BLASTP alignment divided by the protein length of the *C. elegans* query sequence. The heatmaps show the aligned proportion of all BUSCO orthologs, randomly subsampled BUSCO orthologs, and best-reciprocal BLASTP hits. **Table S1.** Summary of genome assemblies. Basic features of nine diplogastrid genomes are shown together with the BUSCO results for the OrthoDB data sets odb10 and odb9 (C:= Complete single copy, D:= Duplicated, F:= Fragmented, M:=Missing - Percentage values are presented as integers. Therefore, values might not always sum up to 100). **Table S2.** Summary of transcriptome assemblies. Basic features of nine transcriptome assemblies are shown together with the BUSCO results for the OrthoDB data sets odb10 and odb9 (C:= Complete single copy, D:= Duplicated, F:= Fragmented, M:=Missing – Percentage values are presented as integers. Therefore, values might not always sum up to 100).

## Data Availability

Genome data is available at the European Nucleotide Archive under the study accession PRJEB27334. All data sets were submitted to WormBase ParaSite and are also available at http://pristionchus.org/download/diplogastrid_annotation_ppcac_v1.tgz. Source code is available at https://github.com/roedelsberg/ppcac.

## Abbreviations

BUSCO: Benchmarking Universal Single Copy Orthologs
ORF: Open Reading Frame
PPCAC: Perl Package for Customized Annotation Computing
