# Supplementary material for: The community-curated Pristionchus pacificus genome facilitates automated gene annotation improvement in related nematodes
Source: BMC Genomics. 2021 Mar 25;22:216. doi: 10.1186/s12864-021-07529-x (PMC7992802; doi:10.1186/s12864-021-07529-x)
Supplement: Supplementary file 1 — Additional file 1: Figure S1. Heuristic approach for reducing the complexity of redundant annotations. A 30kb genomic locus is visualized in the Integrative Genomics Viewer with different tracks showing the exonerate alignments of protein homology data, transcribed ORFs, and the resulting non-redundant gene annotations. The lower plot shows the coverage of exonic features in 100bp windows. First, a 100bp window with maximal coverage is selected. Second, the overlapping gene model with longest ORF is chosen as representative gene model for this locus. Third, all other overlapping gene models are excluded from further analysis. Fourth, the next 100bp window is chosen. This procedure is continued until all 100bp windows have been processed. The final gene models are shown in the track labeled as ‘Non-redundant gene models’. Figure S2. Comparison of normalized bitscores and aligned proportion. a The left heatmap shows the normalized bitscores (bitscore / alignment length) for 1490 BUSCO orthologs derived from a BLASTP search of the C. elegans proteins against annotated protein sets of the ten diplogastrid genomes. The central heatmap shows the data for 101 randomly subsampled BUSCO orthologs. The right heatmap shows the normalized bitscore for 101 putative orthologs that were only predicted based on best-reciprocal BLASTP hits. b The aligned proportion was computed as the length of the BLASTP alignment divided by the protein length of the C. elegans query sequence. The heatmaps show the aligned proportion of all BUSCO orthologs, randomly subsampled BUSCO orthologs, and best-reciprocal BLASTP hits. Table S1. Summary of genome assemblies. Basic features of nine diplogastrid genomes are shown together with the BUSCO results for the OrthoDB data sets odb10 and odb9 (C:= Complete single copy, D:= Duplicated, F:= Fragmented, M:=Missing - Percentage values are presented as integers. Therefore, values might not always sum up to 100). Table S2. Summary of transcriptome assemblie [file 12864_2021_7529_MOESM1_ESM.pdf]

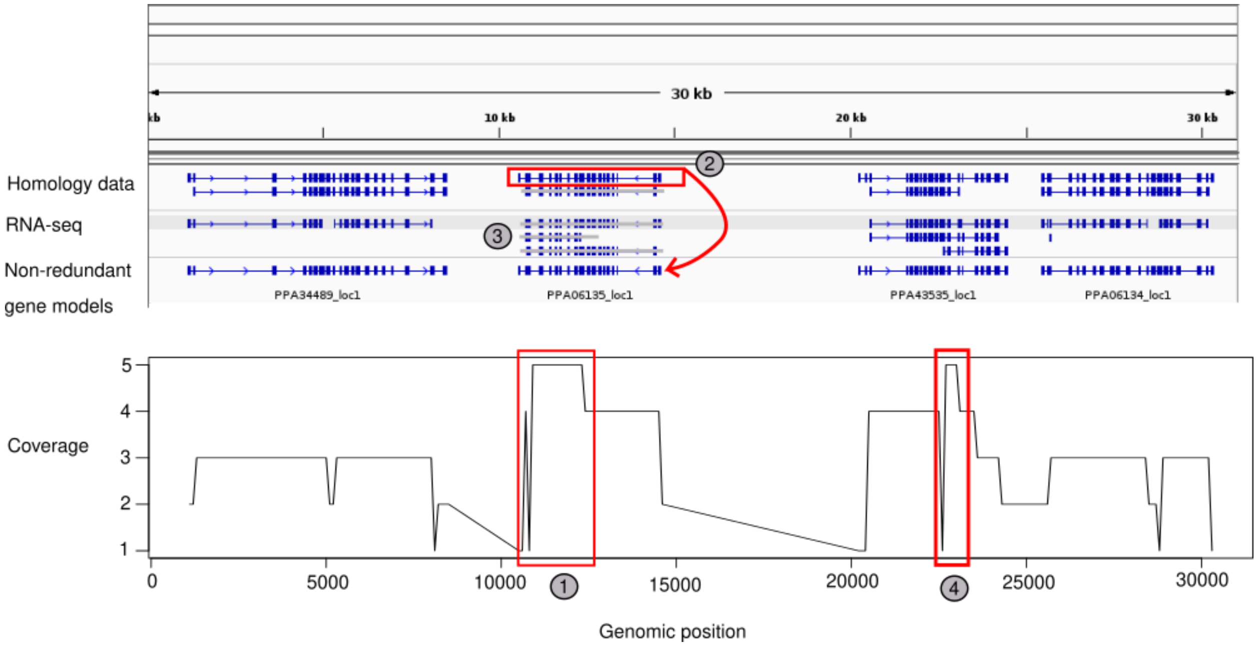

Figure S1 Heuristic approach for reducing the complexity of redundant annotations. A 30kb genomic locus is visualized in the Integrative Genomics Viewer with different tracks showing the exonerate alignments of protein homology data, transcribed ORFs, and the resulting non-redundant gene annotations. The lower plot shows the coverage of exonic features in 100bp windows. First, a 100bp window with maximal coverage is selected. Second, the overlapping gene model with longest ORF is chosen as representative gene model for this locus. Third, all other overlapping gene models are excluded from further analysis. Fourth, the next 100bp window is chosen. This procedure is continued until all 100bp windows have been processed. The final gene models are shown in the track labeled as ‘Non-redundant gene models’.

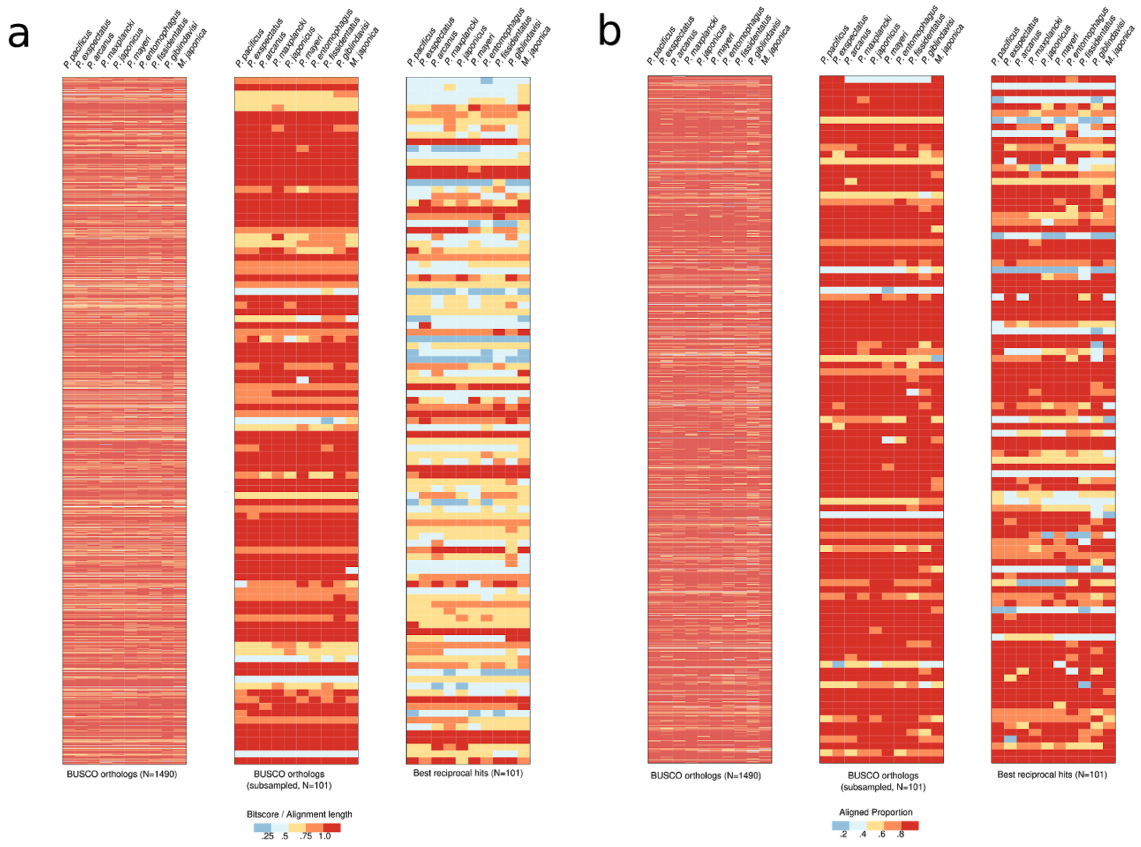

Figure S2 Comparison of normalized bitscores and aligned proportion. a The left heatmap shows the normalized bitscores (bitscore / alignment length) for 1490 BUSCO orthologs derived from a BLASTP search of the *C. elegans* proteins against annotated protein sets of the ten diplogastrid genomes. The central heatmap shows the data for 101 randomly subsampled BUSCO orthologs. The right heatmap shows the normalized bitscore for 101 putative orthologs that were only predicted based on best-reciprocal BLASTP hits. **b** The aligned proportion was computed as the length of the BLASTP alignment divided by the protein length of the *C. elegans* query sequence. The heatmaps show the aligned proportion of all BUSCO orthologs, randomly subsampled BUSCO orthologs, and best-reciprocal BLASTP hits.

Species	European Nucleotide Archive accession	Genome size (MB)	Number of scaffolds	N50 (kb)	BUSCO (%) odb10 C/D/F/M	BUSCO (%) odb9 C/D/F/M
P. exspectatus	GCA_900380275.1	178	4412	142	76/6/2/17	86/5/5/4
P. arcanus	GCA_900490705.1	203	4263	271	76/6/2/17	86/6/4/4
P. maxplancki	GCA_900490775.1	266	69,506	309	72/9/2/17	81/9/6/4
P. japonicus	GCA_900490845.1	223	33,291	448	76/6/2/17	83/7/5/5

P. mayeri	GCA_900490875.1	297	84,599	235	78/3/2/18	83/4/7/7
P. entomophagus	GCA_900490825.1	264	72,722	369	77/3/2/18	83/4/7/6
P. fissidentatus	GCA_900490895.1	247	56,870	443	80/2/2/17	89/1/6/5
Parapristionchus giblindavisi	GCA_900491355.1	201	7303	112	76/2/2/20	77/2/10/11
Micoletzkya japonica	GCA_900490955.1	201	137,965	189	82/2/1/15	85/2/7/7

Table S1 Summary of genome assemblies. Basic features of nine diplogastrid genomes are shown together with the BUSCO results for the OrthoDB data sets odb10 and odb9 (C:= Complete single copy, D:= Duplicated, F:= Fragmented, M:=Missing - Percentage values are presented as integers. Therefore, values might not always sum up to 100).

Species	European Nucleotide Archive accession	Number of sequences	BUSCO (%) odb10 C/D/F/M	BUSCO (%) odb9 C/D/F/M
P. exspectatus	HAKR01000000	102,717	40/44/2/14	46/49/4/1
P. arcanus	HALB01000000	105,577	45/40/2/14	53/41/5/1
P. maxplancki	HAKM01000000	69,790	59/25/2/15	66/27/5/2
P. japonicus	HAKD01000000	64,151	61/22/2/15	70/23/5/2
P. mayeri	HAKW01000000	87,289	52/32/1/15	59/34/5/2
P. entomophagus	HAJU01000000	67,601	59/25/2/15	63/29/6/2
P. fissidentatus	HAKL01000000	60,342	57/23/3/18	66/25/6/4
Parapristionchus giblindavisi	HAJV01000000	49,825	50/24/3/23	56/27/9/9
Micoletzkya japonica	ERX2050362	69,196	61/24/1/14	69/26/4/1

Table S2 Summary of transcriptome assemblies. Basic features of nine transcriptome assemblies are shown together with the BUSCO results for the OrthoDB data sets odb10 and odb9 (C:= Complete single copy, D:= Duplicated, F:= Fragmented, M:=Missing - Percentage values are presented as integers. Therefore, values might not always sum up to 100).
